# Distribution and Effect of Galanin on Gallbladder and Sphincter of Oddi Motility in the Pig

**DOI:** 10.1155/1991/20383

**Published:** 1991

**Authors:** Henrik Harling, Tina Messell, Steen Lindkaer Jensen, Steen Seier Poulsen

**Affiliations:** 1 Department of Surgical Gastroenterology C, Rigshospitalet Institute of Medical Physiology C and Anatomy B the Panum Institute, University of Copenhagen, Copenhagen Department of Surgical Gastroenterology L, Århus Municipal Hospital, University of Århus Denmark; 2 Department of Surgery T KAS Glostrup Ndr. Ringvej Glostrup DK-2600 Denmark

## Abstract

This study was designed to determine the occurrence and topographical distribution of galanin-like immunoreactivity (GAL-LI) in the porcine gallbladder and sphincter of Oddi and to investigate the pharmacologic effect of GAL on gallbladder and sphincter of Oddi motility. By radioimmunoassay the concentration of GAL-LI in the gallbladder was 2.75 ± 0.23, 9.73 ± 1.33 in the common bile duct and 5.10 ± 0.37 in the sphincter of Oddi (pmol/g ± SE). By immunohistochemistry GAL-LI was found exclusively in ganglionic cells and in nerve fibers among the smooth muscle bundles. Gallbladder and sphincter of Oddi pressures were recorded before and during 5-minute local intraarterial infusion of 4, 8, 19, 39, 78 and 194 ng GAL-Kg^-1^-min^-1^ in 12 anaesthetized pigs. GAL in doses ≥ 39 ng.kg^-1^.min^-1^ significantly reduced sphincter of Oddi phasic wave frequency (4.8 ± 0.4 vs. 2.1 ± 0.5; p = 0.004) and sphincter of Oddi motility index (70.2 ± 6.02 vs. 27.7 ± 8.3; p = 0.002) but did not affect gallbladder pressure. We conclude that the distribution of GAL-LI in the sphincter of Oddi and the effect that a pharmacologic dose of GAL has on sphincter of Oddi motor activity, suggests that GAL may be involved in the physiologic control of bile flow in the pig.


*HPB Surgery*, 1991, Vol. 3, pp. 279–289
Reprints available directly from the publisher
Photocopying permitted by license only

© 1991 Harwood Academic Publishers GmbH
Printed in the United Kingdom

### DISTRIBUTION AND EFFECT OF GALANIN ON GALLBLADDER AND SPHINCTER OF ODDI MOTILITY IN THE PIG

HENRIK HARLING,* TINA MESSELL, STEEN LINDKAER JENSEN and STEEN SEIER POULSEN

*Department of Surgical Gastroenterology C, Rigshospitalet, Institute of Medical Physiology C and Anatomy B, the Panum Institute, University of Copenhagen, Copenhagen, Department of Surgical Gastroenterology L, Århus Municipal Hospital, University of Århus, Denmark*

*(Received 3 May 1990)*

This study was designed to determine the occurrence and topographical distribution of galanin-like immunoreactivity (GAL-LI) in the porcine gallbladder and sphincter of Oddi and to investigate the pharmacologic effect of GAL on gallbladder and sphincter of Oddi motility. By radioimmunoassay the concentration of GAL-LI in the gallbladder was 2.75 ± 0.23, 9.73 ± 1.33 in the common bile duct and 5.10 ± 0.37 in the sphincter of Oddi (pmol/g ± SE). By immunohistochemistry GAL-LI was found exclusively in ganglionic cells and in nerve fibers among the smooth muscle bundles. Gallbladder and sphincter of Oddi pressures were recorded before and during 5-minute local intraarterial infusion of 4, 8, 19, 39, 78 and 194 ng GAL - $Kg^{-1}$ - $min^{-1}$ in 12 anaesthetized pigs. GAL in doses ≥ 39 $ng.kg^{-1}.min^{-1}$ significantly reduced sphincter of Oddi phasic wave frequency (4.8 ± 0.4 vs. 2.1 ± 0.5; $p = 0.004$) and sphincter of Oddi motility index (70.2 ± 6.02 vs. 27.7 ± 8.3; $p = 0.002$) but did not affect gallbladder pressure. We conclude that the distribution of GAL-LI in the sphincter of Oddi and the effect that a pharmacologic dose of GAL has on sphincter of Oddi motor activity, suggests that GAL may be involved in the physiologic control of bile flow in the pig.

KEY WORDS: biliary motility, gastrointestinal motility, pigs

Galanin (GAL) is a 29-amino acid residue polypeptide isolated from porcine intestinal extract by Tatemoto *et al.* in 1983[1]. In the intestinal tract galanin-like immunoreactivity (GAL-LI) is found predominantly in neurons of the submucous and myenteric plexus, and large numbers of GAL-LI nerve fibers surround circular smooth muscle bundles[2]. The effect of synthetic porcine GAL on intestinal motor activity is apparently species specific, thus GAL contracts rat intestinal muscle[1,3] and inhibits intestinal contraction in the guinea-pig and dogs[3,4]. In humans intravenous infusion of GAL delays gastric emptying and prolongs intestinal transit time[5]. Therefore a modulatory role of GAL in intestinal motility is likely, and it seems conceivable that biliary motility could also be influenced by GAL.

The present study was undertaken to examine the occurrence and distribution of GAL-LI in the porcine gallbladder and biliary tract. We also studied the effect of GAL on gallbladder and sphincter of motility in anesthetized pigs.

Address for correspondence: Henrik Harling, Department of Surgery T, KAS Glostrup, Ndr. Ringvej, DK-2600 Glostrup, Denmark.

### MATERIALS AND METHODS

### *Animals and Preparation*

Danish LYY-strain pigs weighing 28–32 kg were used. The pigs were denied food for 24 h but had free access to drinking water. After premedication with ketamin chloride (Ketalar(R), Parke Davis) 10 mg/kg the pigs were anaesthetized with 1.5% halothane. Following intubation intravenous chloralose (no 2420 Merck, Darmstadt, FRG) 100 mg/kg was substituted for halothane, and anaesthesia was maintained with chloralose and $N_2O$. The animals were placed on a 38°C heat blanket, and intravascular volume and blood pressure was maintained throughout the experiment by intravenous infusion of 6 ml/min of lactated Ringers's solution. An abdominal midline incision was used. Full wall biopsies from the gallbladder, common bile duct, sphincter of Oddi and duodenum were excised for analysis of occurrence of galanin (n = 5).

The effect of GAL on motility was examined in 12 pigs. After removal of the spleen, an open-tip catheter for infusion of GAL was placed in the common hepatic artery (Figure 1). The gallbladder bile was aspirated and a polyethylene 5 Fr catheter was inserted into the gallbladder fundus through the aspiration needle hole and secured with a suture. The common bile duct was opened in the mid portion, and a catheter was inserted proximally for drainage of gallbladder perfusate and hepatic bile. The gallbladder catheter was perfused at 0.1 ml/min with distilled water via a hydraulic capillary infusion system, and pressures were transmitted to transducers (American Edwards Laboratories RH3WDPT2AV). With this perfusion system, the resting gallbladder pressure was stable in the range of 7–10 mmHg. Thus a potential influence on the sphincter of Oddi by the cholecysto-sphincter of Oddi reflex[6] could be disregarded. A second choledochotomy was made more distally, and a 5 Fr pressure-monitored perfusion catheter was inserted through the distal common bile duct into the duodenum. Distally the catheter was mounted with a metal tip bearing 3 side holes located 120° apart. The side holes were perfused at 0.1 ml/min as mentioned previously.

The catheter was then pulled back until the side holes entered the high-pressure zone of sphincter of Oddi. A duodenal tube decompressed the stomach, drained the perfusate of the distal common bile duct, and obviated potential influence of the gastro — sphincter of Oddi reflex[7]. Gallbladder and sphincter of Oddi pressures were recorded simultaneously on a multi-channel writer (Elema Schonander 81, Sweden) for a period of 30 min to ensure that the system had stabilized. Intraarterial infusions of synthetic porcine GAL (Bachem, Bubendorf, Switzerland) dissolved in 0.9% NaCl containing 67 nmol human serum albumin/l at 4, 8, 19, 39, 78 and 194 ng - $kg^{-1}$ - $min^{-1}$ were given in a random order, each for a period of 5 min. Each infusion period was preceded by a 15-min control period, during which phasic wave activity returned to preinfusion levels. For calculations only the last minute of infusion and control periods were used. Sphincter of Oddi recordings were analysed for baseline pressure, and for frequency and amplitude of phasic waves. A sphincter of Oddi motility index was calculated from the product of the frequency and the mean amplitude of phasic waves for that minute according to Muller *et al.*[8]. The preinfusion gallbladder pressure was compared with maximal pressure changes achieved during GAL infusions.

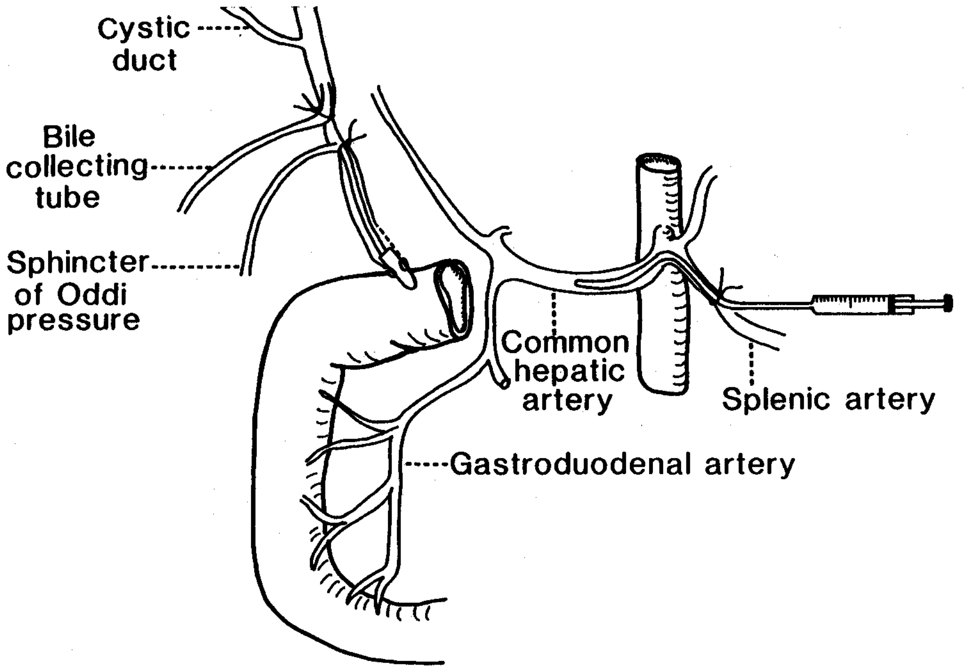

**Figure 1** Drawing of the preparation showing the position of the GAL infusion catheter into the arterial supply of the sphincter of Oddi.

*Extraction, Radioimmunoassay and Immunohistochemistry*

Tissue specimens were frozen immediately, stored at −20°C and weighed before extraction in boiling, redistilled water (5 ml/g wet weight). After homogenization and centrifugation (1500 g at 4°C for 15 min), the supernatant was decanted and the residue resuspended and homogenized in 1 M acetic acid. The combined supernatants were centrifuged and evaporated to dryness in vacuo. The samples were reconstituted and diluted in assay buffer (0.05 mol/l sodium phosphate, pH 7.5, containing 1g/l human serum albumin, 0.1 mol/l sodium chloride and 20 mmol/l EDTA). GAL was measured by radioimmunoassay, and antisera were raised in rabbits. The assay procedure has been reported in detail previously[9]. Tissue for immunohistochemical investigation was obtained by perfusion through the intraarterial cannula with saline for a few minutes to remove blood. Following perfusion with 4% paraformaldehyde in 0,1 M sodium phosphate, pH 7,4 for 10 min, specimens from the gallbladder, common bile duct and sphincter of Oddi were removed and postfixed in the same solution for 24 hours. For cryosections the specimens were transferred to 20% sucrose in 0,1 M phosphate buffer for 24 hours. Tissue blocks were frozen in melting Freon 12 and cut into sections of approximately 10$\mu$. Immunoreactivity was visualized by means of the peroxidase-antiperoxidase (PAP) technique[10] using swine antirabbit IgG and peroxidase antiperoxidase (DakoPatt, Denmark) and diaminobenzidine for staining. The antiserum against galanin (2185–1) was diluted 1:400 and 1:1600 with 0, 1M

phosphate buffer, and the cryostat sections were incubated for 23 hours at 4°C and 1 h at room temperature.

The antiserum absorbed with an excess of galanin (10 nmol/ml of antiserum diluted 1:1600) was used as control for the specificity of the immunostaining, and the antiserum was replaced by non-immune serum as control for non-specific staining.

### *Statistical Evaluation*

The Wilcoxon test was used to compare sphincter of Oddi phasic wave motility index, frequency, amplitude, baseline pressure, and gallbladder pressure during control and GAL infusion periods. Student's unpaired t test was used to compare control data before GAL infusions. P-values less than 0.05 were considered significant.

**Table 1** Concentration of GAL-LI in porcine tissue specimens (pmol/g, mean ± SE, n = 5).

| | |
|---|---|
| Gallbladder | 2.75±0.23 |
| Common bile duct | 9.73±1.33 |
| Sphincter of Oddi | 5.10±0.37 |
| Duodenum | 3.23±0.43 |

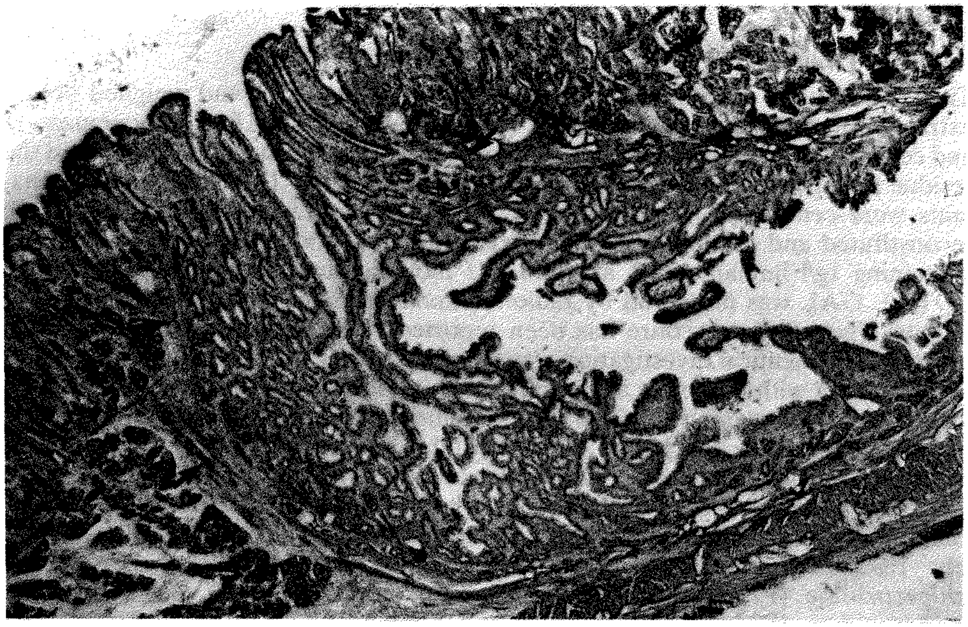

**Figure 2** Low magnification showing the part of the duodenum where the common bile duct traverse the duodenal wall to open into the papilla of Vater. The sphincter, indicated by arrows, contains delicate bundles of smooth muscles. (Cryostat section, × 50)

### RESULTS

### *Occurrence and Distribution of GAL-LI*

Using the method of radioimmunoassay, GAL-LI was found in all extracts of the pig gallbladder and biliary pathways (Table 1). Tissue for immunohistochemistry was taken from the papilla of Vater as shown in Figure 2. GAL-LI nerve fibers were distributed in close relation to the smooth muscle bundles in the sphincter of Oddi (Figure 3), and GAL-LI nerve cell bodies were regularly observed in relation to the sphincter of Oddi (Figure 4). In the gallbladder fine GAL-LI nerve fibers were found in the wall (Figure 5). GAL-LI nerve fibers were very sparse and nerve cells were not observed in the common bile duct.

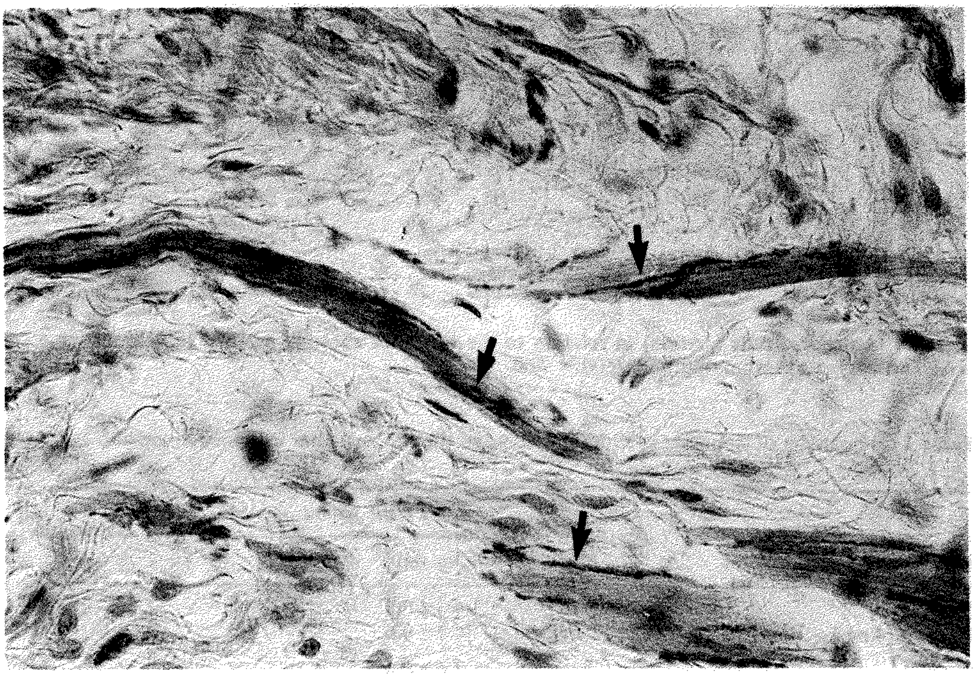

**Figure 3** GAL-LI nerve fibers (arrows) in close relation to the delicate bundles of smooth muscle fibers from the sphincter of Oddi. Antiserum diluted 1:1600 × 520

### *Gallbladder Pressure*

The intragallbladder pressure was not influenced by infusion of 4 – 194 ng GAL - $kg^{-1}$ $min^{-1}$.

### *Sphincter of Oddi Motility*

The baseline pressure of 5.9 ± 0.5 mmHg decreased slightly, but not significantly during infusions of GAL at doses ≥ 39 ng-$kg^{-1}$ $min^{-1}$. As shown in Table 2 the

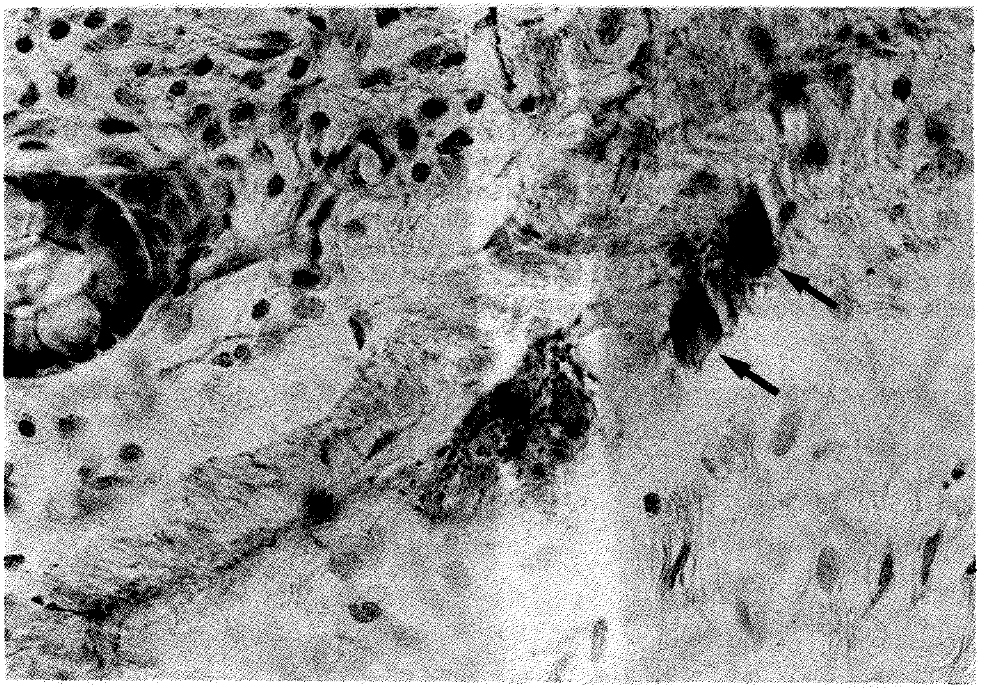

**Figure 4** Ganglionic cells (arrows) in relation to the sphincter showing positive immunoreaction for GAL (arrows). Antiserum diluted 1:1600 × 460

**Table 2** Comparison of sphincter of Oddi phasic waves before and after infusions of galanin

| | *SO Phasic waves* | |
|---|---|---|
| *Local intraarterial infusion* | *No./min* | *Ampl, mmHg* |
| Control | 4.4 ± 0.8 | 14.3 ± 6.3 |
| Galanin (4 ng·kg$^{-1}$·min$^{-1}$) | 4.2 ± 0.6 | 14.9 ± 6.6 |
| Control | 4.6 ± 0.6 | 15.3 ± 3.3 |
| Galanin (8 ng.kg$^{-1}$ min$^{-1}$.) | 4.0 ± 0.6 | 15.1 ± 3.1 |
| Control | 4.6 ± 0.6 | 17.7 ± 3.2 |
| Galanin (19 ng.kg$^{-1}$ min$^{-1}$.) | 4.2 ± 0.4 | 16.3 ± 2.9 |
| Control | 4.6 ± 0.4 | 16.4 ± 3.0 |
| Galanin (39 ng.kg$^{1}$.min$^{-1}$) | 3.1 ± 0.5* | 11.1 ± 2.1 |
| Control | 4.8 ± 0.4 | 15.7 ± 2.4 |
| Galanin (78 ng.kg$^{-1}$.min$^{-1}$) | 2.1 ± 0.5* | 8.5 ± 2.2* |
| Control | 3.8 ± 0.3 | 15.1 ± 3.1 |
| Galanin (194 ng.kg$^{-1}$.min$^{-1}$) | 2.2 ± 0.5* | 9.8 ± 2.5* |

Values are means ± SE. SO, sphincter of Oddi, No, wave frequency Ampl, mean amplitude/min. * $P < 0.05$

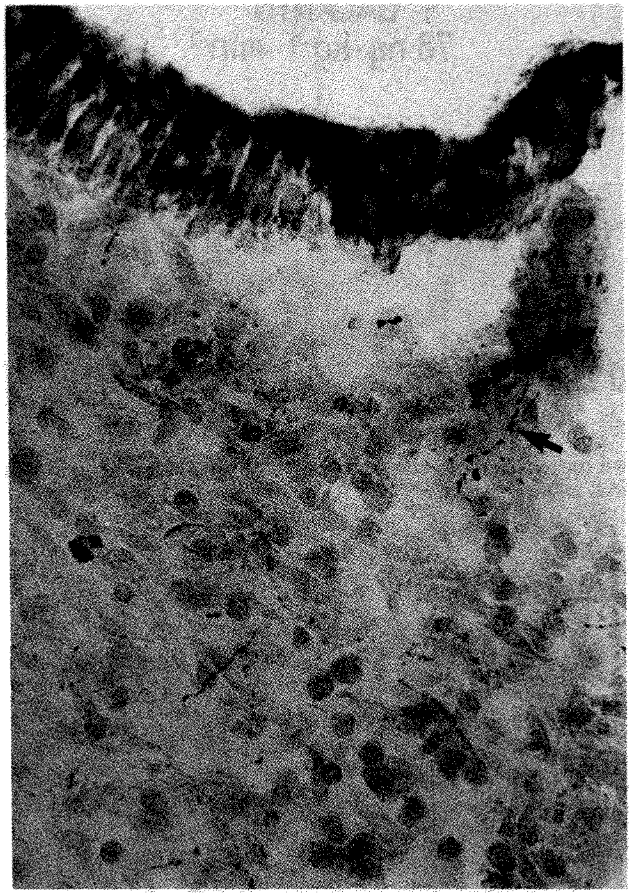

**Figure 5** Fine GAL-LI nerve fiber (arrow) in the wall of the gallbladder. Antiserum diluted 1:1600. × 520

phasic wave frequency and amplitude decreased significantly with increasing doses of GAL. An example of the changes produced in sphincter of Oddi activity by infusion of GAL at 78 $ng\text{-}kg^{-1}\text{-}min^{-1}$ is presented in Figure 6. Considering sphincter of Oddi motility index, preinfusion control data did not differ significantly. The motility index decreased significantly during infusion of GAL doses $\geq$ 39 $ng\text{-}kg^{-1}\text{-}min^{-1}$ (Figure 7).

### DISCUSSION

The musculature of the sphincter of Oddi differs both anatomically and embryologically from that of the surrounding intestinal musculature, and the sphincter

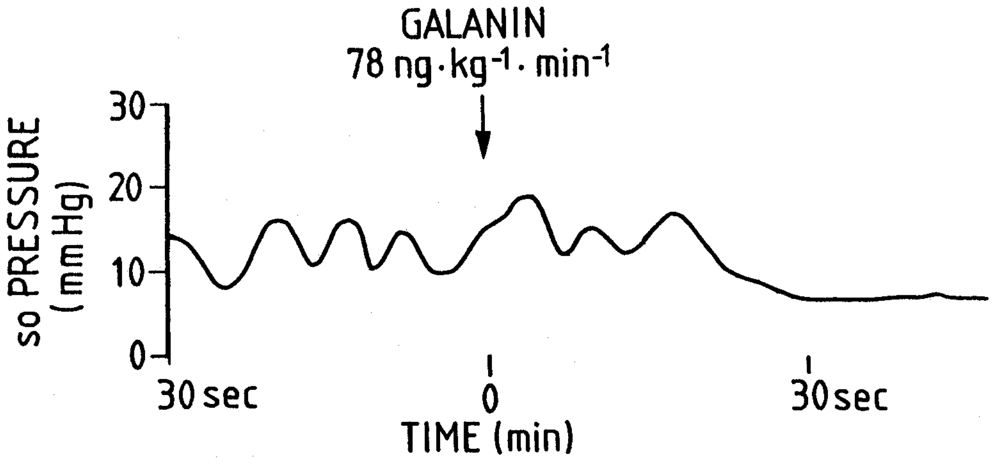

**Figure 6** A redrawn tracing showing reduction in sphincter of Oddi motility in response to intraarterially infusion of GAL in one experiment.

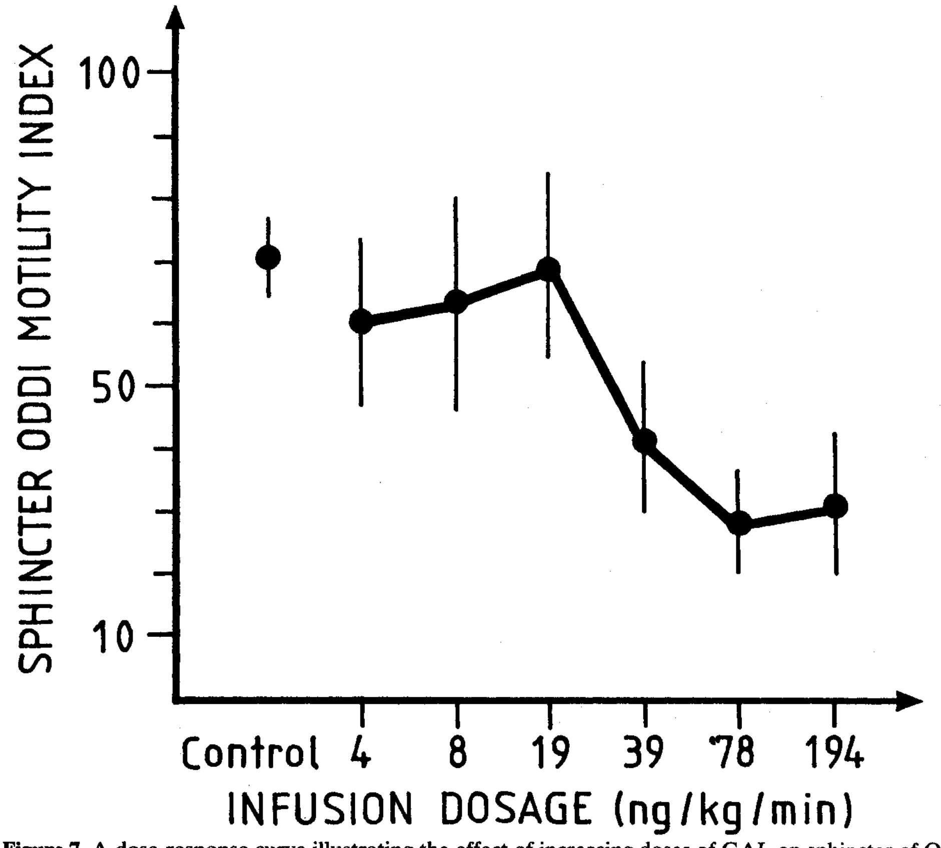

**Figure 7** A dose-response curve illustrating the effect of increasing doses of GAL on sphincter of Oddi motility index. Control data are means of combined preinfusion periods. GAL doses $\geq$ 39 ng-kg$^{-1}$-min$^{-1}$ reduced the index significantly.

functions independently of duodenal muscle activity[11]. The action of the sphincter is to adjust flow from the common bile duct into the duodenum. Thus, in humans and in the opossum bile flows passively into the sphincter segment during sphincter diastole and is expelled into the duodenum by sphincter contraction[11,12]. Biliary tract motility and transsphincteric flow is modified by several hormones[8,13] and an intrinsic ganglionated nerve plexus[14].

Cai *et al.*[15] have documented the occurrence and distribution of the neural peptides vasoactive intestinal polypeptide (VIP), substance P, met-enkephalin and bombesin in the gallbladder and biliary pathways of the guinea pig, and it has been speculated that the inhibitory nonadrenergic, noncholinergic neurotransmitter is VIP[13]. However, bombesin and enkephalines are other candidate agents[13,16].

Since the discovery of GAL several studies have documented its modulatory effects in intestinal motility, whereas a possible role in control of biliary motility has not been elucidated previously. Ideally, pharmacologic experiments should be performed in the homologous species, and because the amino acid sequence of synthetic GAL is porcine we chose the pig for examination. By radioimmunoassay GAL-LI was found in both gallbladder and biliary pathways, but contrary to the observation regarding VIP[17], a rich GAL supply is apparently not characteristic of sphincters. Thus the quantity of GAL-LI in the sphincter of Oddi was about half of that in the common bile duct, a situation much the same as in the lower esophageal sphincter compared to the mid esophageus[9], and the pyloric channel compared to the antrum (Harling *et al.*, unpublished observations). The presence of GAL-LI exclusively in ganglionic cells and in nerve fibers among the smooth muscle bundles of the sphincter of Oddi is consistent with the pattern seen in other parts of the intestinal tract in general, and with the report of Gonda *et al.*[18] on the dog sphincter of Oddi in particular.

In the present analysis of the resting motility pattern, GAL decreased sphincter of Oddi motor activity, specifically by reducing frequency and amplitude of phasic contractions. The baseline pressure decreased only slightly, but as the pressure measured only 5.9 mmHg in this animal model a significant pressure reduction could be hard to demonstrate. Similarly, the lack of an GAL effect on gallbladder motility should be interpreted with caution. Apparently GAL is not a stimulator of gallbladder motility, but an inhibitory action might have been more easily disclosed in a stimulated set-up.

In conclusion, the inhibitory effect of GAL on sphincter of Oddi motility is consistent with other known inhibitory actions of GAL on intestinal motility. However the physiologic significance of our observations must await future studies of GAL's effect on bile flow through the sphincter.

*Acknowledgments*

This work was supported by The Danish Hospital Foundation for Medical Research. Region of Copenhagen, The Faroe Islands and Greenland (27/88).

The technical assistance of Sørn Haagen Nielsen is gratefully acknowledged.

*References*

1. Tatemoto, K., Rökaeus, Å., Jörnvall, H., McDonald, T.J. and Mutt, V. (1983) Galanin- a novel biologically active peptide from porcine intestine. *FEBS Lett.*, **164**, 124–128
2. Melander, T., Hökfelt, T., Rökaeus, Å., Fahrenkrug, J., Tatemoto, K. and Mutt, V. (1985)

Distribution of galanin-like immunoreactivity in the gastro-intestinal tract of several mammalian species. *Cell Tissue Res.*, **239**, 253–270
3. Ekblad, E., Håkanson, R., Sundler, F. and Wahlestedt, C. (1985) Galanin: neuromodulatory and direct contractile effects on smooth muscle preparations. *Br. J. Pharmacol.*, **86**, 241–246
4. Fox, J.E.T., McDonald, T.J., Kostolanska, F. and Tatemoto, K. (1986) Galanin: an inhibitory neural peptide of the canine small intestine. *Life Sciences*, **39**, 103–110
5. Bauer, F.E., Zintel, A., Kenny, M.J., Calder, D., Ghatel, M.A. and Bloom, S.R. (1989) Inhibitory effect of galanin on postprandial gastrointestinal motility and gut hormone release in humans. *Gastroenterology*, **97**, 260–264
6. Muller, E.L., Lewinski, M.A. and Pitt, H.A. (1984) The cholecysto-sphincter of Oddi reflex. *J. Surg. Res.*, **36**, 377–383
7. Webb, T.A., Lillemoe, K.D. and Pitt, H.A. (1988) The gastro-sphincter of Oddi reflex. *Am. J. Surg.*, **155**, 193–198
8. Muller, E.L., Grace, P.A., Conter, R.L., Roslyn, J.J. and Pitt, H.A. (1987) Influence of motilin and cholecystokinin on sphincter of Oddi and duodenal motility. *Am. J. Physiol. (Gastrointest Liver Physiol 16)*, **253**, G679–G683
9. Harling, H., Messell, T., Jensen, S.L., Holst, J.J. and Poulsen, S.S. (1989) Occurrence, distribution and motor effects of galanin in the porcine lower esophageal sphincter. *Digestion*, **42**, 151–157
10. Sternberger, L. (1974) *Immunohistochemistry*, pp. 129–171 New York: Prentice Hall, Englewood Cliffs
11. Toouli, J. (1984) Sphincter of Oddi motility. *Br. J. Surg.*, **71**, 251–256
12. Toouli, J., Dodds, W.J., Honda, R., Sarna, S., Hogan, W.J., Komarowski, R.A., Linehan, J.H. and Arndorfer, R.C. (1983) Motor function of the opossum sphincter of Oddi. *J. Clin. Invest.*, **71**, 208–220
13. Ryan, J.P. (1987) Motility of the gallbladder and biliary tree. In: *Physiology of the gastrointestinal tract*, 2nd ed. pp. 709–714 Leonard R. Johnson (Ed). New York: Raven Press
14. Furness, J.B. and Costa, M. (1987) The enteric nervous system, 187. London: Churchill Livingstone
15. Cai, W., Gu, J., Huang, W., McGregor, G.P., Ghatei, M.A., Bloom, S.R. and Polak, J.M. (1983) Peptide immunoreactive nerves and cells of the guinea pig gall bladder and biliary pathways. *Gut*, **24**, 1186–1193
16. Persson, C.G.A. (1976) Inhibitory innervation of cat sphincter of Oddi. *Br. J. Pharmacol.*, **58**, 479–482
17. Alumets, J., Fahrenkrug, J., Håkanson, R., Schaffalitzky de Muckadell, O., Sundler, F. and Uddman, R. (1979) A rich VIP nerve supply is characteristic to sphincters. *Nature*, **280**, 155–156
18. Gonda, T., Daniel, E.E., McDonald, T.J., Fox, J.E.T., Brooks, B.D. and Oki, M. (1989) Distribution and function of enteric Gal-IR nerves in dogs: comparison with VIP. *Am. J. Physiol., (Gastrointest Liver Physiol 19)*, **256**, G884–G896

*(Accepted by S. Bengmark 2 July 1990)*

### INVITED COMMENTARY

The authors have described the presence of intrinsic enteric GAL-LI neurons in the biliary tract of the pig; they have further demonstrated that an infusion of porcine GAL via the hepatic artery had inhibitory motor effects on the sphincter of Oddi. These two observations lead to the possible conclusion that the GAL-LI neurons in the porcine sphincter of Oddi are inhibitory motor neurons and may participate in the regulation of sphincter motor function.

However, the results of the GAL infusion studies must be interpreted with caution. GAL has not been established as a neurotransmitter and in the present experiments the site of action of GAL has not been determined. For example,

GAL may have acted directly on the muscle, via other intrinsic neurons or via local release of a hormone.

Nonetheless, this study expands on an important new area of research — the role of the enteric nervous system in modulating biliary tract function. Over the last decade a number of neurally mediated biliary tract reflexes have been described. Thune and associates observed a decrease in resistance to flow across the feline sphincter of Oddi in response to an increase in pressure in the proximal bile ducts and gallbladder[1]. Cholecystectomy and division of the nerves at the junction of the cystic duct and bile duct abolished the inhibitory reflex[2]. It was postulated that the neural pathways mediating the inhibitory influence were interrupted during the removal of the gallbladder.

Sphincter of Oddi motility dysfunction produces post-cholecystectomy biliary pain[3]. Interference with the biliary tract neural control mechanisms may be a factor in the aetiology of this condition. It is only after the results of correlated anatomical and functional studies are obtained that the role of the enteric nervous system in normal and disordered function may be established.

R.T.A. Padbury
Department of Surgery
Flinders Medical Centre
Adelaide, Australia

*References*

1. Thune, A., Thornell, E. and Svanvik, J. (1986) Reflex regulation of flow resistance in the feline sphincter of Oddi by hydrostatic pressure in the biliary tract. *Gastroenterology*, **91**, 1364–1369
2. Thune, A., Jivegard, L., Conradi, N. and Svanvik, J. (1988) Cholecystectomy in the cat damages pericholedochal nerves and impairs reflex regulation of the sphincter of Oddi. A mechanism for post-cholecystectomy biliary dyskinesia. *Acta Chir. Scand.*, 191–194
3. Toouli, J. (1990) Sphincter of Oddi Dysfunction. Is it a clinically relevant condition? *Br. J. Surg.*, **77**, 723–724

